# Risk‐Taking Facilitates Implicit Learning in Young Adults

**DOI:** 10.1002/brb3.70409

**Published:** 2025-03-13

**Authors:** Amanda Cremone‐Caira, Melissa A. St. Hilaire

**Affiliations:** ^1^ Department of Psychology Merrimack College North Andover Massachusetts USA; ^2^ Department of Computer and Data Sciences Merrimack College North Andover Massachusetts USA

**Keywords:** cognition, executive function, implicit learning, reward processing, risk‐taking

## Abstract

**Introduction:**

Risk‐taking is associated with dynamic outcomes, including psychopathology and types of learning related to adaptive behaviors. The goal of the current study was to (1) evaluate risk‐related learning in a sample of neurotypical young adults and (2) determine how risk‐taking related to motivation and emotional processing (as measured by BIS/BAS Scales).

**Methods:**

Fifty‐eight young adults (*M*_age_ = 19.66 years, SD = 1.43 years; 74% female) completed the Balloon Emotional Learning Task (BELT) and the BIS/BAS to measure risk‐taking tendencies and motivation and emotional processing, respectively.

**Results:**

Generalized linear mixed models indicate that participants learned to make more advantageous decisions as they engaged in risk‐taking behaviors during the BELT. Risk‐taking outcomes were positively correlated with self‐report of participant's persistent pursuit of goals as measured by the BAS Drive Scale, although these findings were no longer significant after correcting for multiple comparisons.

**Conclusions:**

Together, these results suggest that, in some contexts, risk‐taking may support learning and goal‐directed behaviors in young adults. These findings have notable implications in improving educational and professional outcomes.

## Graphical abstract

Young adults demonstrated improved decision‐making as they engaged in risk‐taking behaviors during the Balloon Emotional Learning Task (BELT), suggesting that risk‐taking enhances learning. Risk‐taking was also linked to goal‐pursuit motivation, measured by the BAS Drive Scale, although this relationship was not significant after multiple comparison corrections. Overall, these findings indicate potential benefits of risk‐taking in fostering adaptive learning, with implications for education and professional development.

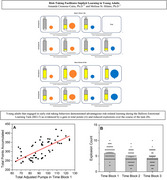

## 1 Introduction

Risk‐taking refers to behavior that results in uncertain outcomes (Mohr et al. 2010). According to models of decision theory (largely based on von Neumann and Morgenstern 1944), risk‐taking behaviors are often conceptualized by two opponent patterns. “Risk aversion” is marked by the avoidance of potential gain to maintain stability. In contrast, “risk‐seeking” is marked by avoidance of stability to increase potential gains. Traditionally, risk‐seeking behaviors are associated with maladaptive outcomes including, but not limited to, impulsivity and poor decision making (Cho et al. 1999; Del Popolo et al. 2024; Gong et al. 2022) as well as psychopathology (e.g., attention‐deficit/hyperactivity disorder, addictive behaviors) (Boyer 2006; Mishra et al. 2010; Petry 2001; Secades‐Villa et al. 2016). Consequently, risk‐aversion is often considered to be adaptive (note, however, that clinically elevated levels of risk aversion may also be indicative of anxiety disorders, e.g., Eisenberg et al. 1998; Hartley and Phelps 2012; Maner et al. 2007). However, accumulating evidence suggests the outcomes of risk‐seeking behaviors are context‐dependent and that, in some cases, may support learning.

Recent research using the Balloon Analog Risk Task (BART) provides evidence of risk‐related learning. The BART is an objective assessment of risk‐taking tendencies where risk‐seeking behaviors are rewarded up to a certain threshold, beyond which additional risk leads to negative outcomes (Lejuez et al. 2002). Using a unique adaptation of the BART, Meshi et al. (2020) reported that excessive social media use was associated with heightened risk‐aversion, but only after participants experienced loss. These results demonstrate probabilistic reversal learning, as risk‐taking behavior changed depending on a learned reward contingency (high‐risk vs. low‐risk scenarios). Likewise, Sebri et al. (2023) performed trial‐level analysis of BART data and reported changes in risk‐taking behaviors based on prior outcomes (specifically, gains and losses), further illustrating risk‐related learning.

Adapted from the BART, the Balloon Emotional Learning Task (BELT) is a computerized task that measures individual differences in learning through risk‐taking. In contrast to traditional risk‐taking tasks (e.g., the BART and Iowa Gambling Task), the BELT includes variable and stable conditions that allows the tracking of condition differentiation that implicitly facilitates risk‐related learning. Using the BELT, Humphreys et al. (2013) reported that individuals who engaged in high rates of sensation‐seeking behaviors *and* showed high levels of sensitivity to learning from those risks, performed significantly better on the task than participants who engaged in sensation‐seeking behaviors but did not learn, and participants who were otherwise risk‐averse. Thus, risk‐seeking behaviors led to improved task performance only when participants learned from the risks previously taken.

To build on the work of Humphreys et al. (2013), further research is needed to bridge the gap between objective measures of risk‐taking and their associations with adaptive behaviors in adult populations. To date, a limited number of studies have empirically evaluated the associations between risk‐taking tendencies and adaptive behaviors in adult samples (Fischer and Smith 2004; Hansen and Breivik 2001; Wood et al. 2013; Blair et al. 2018).

Further, most of the existing literature is limited to subjective assessments such as participant self‐report (Fischer and Smith 2004; Hansen and Breivik 2001; Wood et al. 2013). In this literature, relations between risk‐taking and adaptive outcomes were often examined very narrowly (e.g., in the context of certain personality traits or behavioral constructs; Fischer and Smith 2004; Wood et al. 2013; Blair et al. 2018; Kwon et al. 2022) or not at all (Hansen and Breivik 2001). As such, additional research assessing objective measures of risk‐taking behavior in relation to specific adaptive behaviors in adults is sorely needed.

Work in adolescents suggests that risks which (1) benefit an individual's well‐being, (2) carry potential costs that do not harm an individual's health or well‐being, and (3) are socially acceptable, can positively influence developmental outcomes (see Duell and Steinberg 2019 for review). Consider for example, the choice to initiate a new friendship, try a new dish at a restaurant, or enroll in a challenging course. All these scenarios are inherently risky as the consequences are unknown—however, those consequences come at a minimal cost and may result in advantageous outcomes.

Although much of the existing literature in adult populations highlights the potential negative outcomes associated with risk‐taking behaviors, nuanced theoretical models account for the complexities of risk‐taking and emphasize the importance of examining contexts where such behaviors might yield adaptive benefits. For example, Ernst's Triadic Model offers valuable insights into the neural and motivational processes underlying these behaviors. The Triadic Model (Ernst et al. 2006; Ernst 2014) outlines three key neural systems—approach (reward‐driven, primarily involving the nucleus accumbens), avoidance (harm‐avoidant, centered around the amygdala), and regulation (mediated by the prefrontal cortex)—and their interactions in motivated behaviors. This model can be adapted to examine how these systems influence relations between risk‐taking behaviors and motivation and emotional processes in adults as the neural systems involved are presumed more mature during adulthood.

Relatedly, research indicates that the outcomes derived from different risk‐taking measures vary with age. For example, Braams et al. (2015) reported that performance on the BART varied in an adolescent sample, whereas a self‐reported measure of risk‐taking tendencies—namely, the Behavioral Inhibition System/Behavior Activation System (BIS/BAS) Scales—was stable across adolescence and early adulthood (Braams et al. 2015; Takahashi et al. 2007). The BIS/BAS Scales (Carver and White 1994) measure generalizable risk‐taking tendencies related to an individual's inherent motivational or emotional responses. Interestingly, however, Braams et al. (2015) reported a low correlation between the BART and BIS/BAS, further demonstrating a difference in measurement in this age group. In contrast, Li et al. 2019 reported that approach motivation was associated with increased risk‐taking behaviors in adolescents. Taken together, these findings suggest that objective and subjective measures may estimate different aspects of individual differences in risk‐taking behavior in adolescents. Whether relations between objective and self‐reported risk‐taking outcomes are evident in adults, when the neural systems underlying approach and avoidance are matured, warrants further research.

### 1.1 Current Study

The goals of the current study were two‐fold. First, we aimed to evaluate risk‐related learning in a sample of neurotypical young adults. It was hypothesized that participants who engaged in early, exploratory risk‐taking behaviors would demonstrate improved performance over the course of the task. Second, we were interested in how risk‐taking outcomes correlate to individual differences in motivation and emotional processing (as measured by BIS/BAS Scales) in this sample. Work in adolescents indicates that approach motivation positively correlates with risk‐taking behavior (Li et al. 2019). Moreover, motivation and positive emotional state are reported to positively relate to risk‐taking behaviors in young adults (Cooper et al. 2000; Leikas et al. 2009). As such, we hypothesized that risk‐taking behavior would positively correlate with motivation and positive emotional state in our young adult sample.

## 2 Materials and Methods^1^

### 2.1 Participants

Participants were recruited through the student research participation platform, SONA, facilitated by the Department of Psychology at Assumption University. All SONA participants completed a prescreening questionnaire to evaluate study eligibility. Participants with a history or current diagnosis of a self‐reported addictive behavior disorder and/or color blindness were ineligible to participate in this study. Data were obtained from 58 participants (*M*_age_ = 19.66 years, SD = 1.43 years; 74% female). In this sample, 74.1% of participants were White/Caucasian, 8.6% of participants were Black/African American, 5.2% of participants were Chinese, and 6.9% of participants identified as “other” race.^2^

### 2.2 Measures

#### 2.2.1 Balloon Emotional Learning Task (BELT)

**FIGURE 1:**
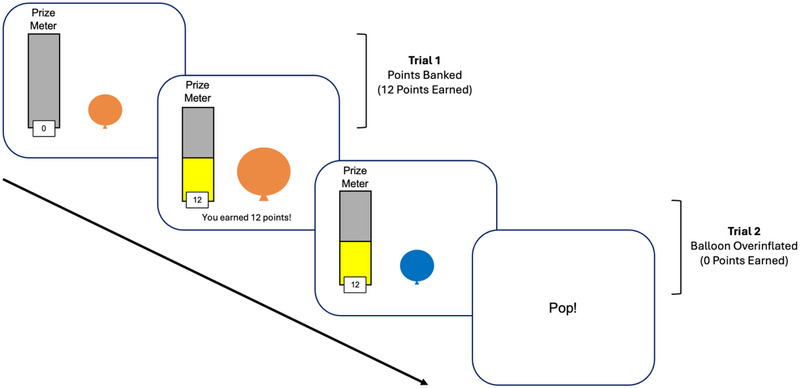
Stimulus presentation during the Balloon Emotional Learning Task (BELT).

The BELT is an exploration‐based, implicit learning task that measures differences in risk‐taking behavior (Humphreys et al. 2013; Figure 1). During the task, participants pressed a button on a keyboard to inflate images of balloons displayed on the computer screen. Each time the participant pressed a button to inflate the balloon, they earned one point (i.e., one pump = one point). The goal of the task was to earn as many points as possible. Importantly, however, participants were informed that balloons would pop if over‐inflated and that not all balloons popped at the same point. Participants were able to “bank” (i.e., save) their points after at least one pump. When points were saved, participants heard a tone that signified success, and the number of pumps were converted to “points” on a visible prize meter which displayed the total number of points accumulated throughout the task. The next trial then began. If a participant over‐inflated a balloon, participants heard a tone indicative of an explosion and the word “Pop!” appeared on the screen. No points were added to the prize meter and the next trial began.

Balloons were shown in three different colors that, unbeknownst to the participant, determined the explosion rate of that color balloon. Two of the colors represented stable conditions in which the explosion rate is fixed at a certain short number of pumps (e.g., 6 pumps before explosion) or a certain long number of pumps (e.g., 18 pumps before explosion). The third color represented a variable condition in which the explosion rate was variable (e.g., explosion occurred variably after either 6, 12, or 18 pumps). The different colored balloons were presented equally across 54 trials (18 trials per balloon type) and blocks of the task (18 trials per block).

The two stable conditions allowed for direct examination of learning across the task, as participants should learn which colored balloons to avoid pumping (low explosion rate) and continue pumping (high explosion rate) using feedback from previous trials. Likewise, the variable condition provided an opportunity to evaluate risk‐taking propensity in the context of uncertainty. Consistent with Humphreys et al. (2013), several outcome measures were derived from the BELT including

1. **Pumps** as a measure of general risk‐taking (more pumps = more risk‐taking)
2. **Adjusted pumps** as a measure of successful risk‐taking (number of pumps excluding balloons that exploded; adapted from the BART (Lejuez et al. 2002))
3. **Post‐explosion pumps** as a measure of sensitivity to negative feedback
4. **Optimal pumps** as a measure of implicit learning (difference between number of pumps and balloon durability).

#### 2.2.2 Behavioral Inhibition System/Behavioral Activation System (BIS/BAS) Scale

The BIS/BAS Scales measure individual differences in two opponent processes related to motivation and emotional processing (Carver and White 1994). Participants use a four‐point response scale to rate the degree to which they agree or disagree with 24 items, where responses of “1” represent strong agreement (“very true for me”) and responses of “4” represent low agreement (“very false for me”). All but two items are reverse scored (e.g., responses of “4” become “1”). Responses across subsets of items are then averaged to create scales of two dimensions of motivation and emotional processing.

The behavioral inhibition system (BIS) scale measures negative emotional responses to punishment (e.g., “Criticism or scolding hurts me quite a bit.”) whereas the behavioral activation or approach system (BAS) scale measures positive emotional responses to reward. BAS items are further categorized by behaviors related to Reward Responsiveness (e.g., “When I'm doing well at something I love to keep at it.”), Drive (e.g., “I go out of my way to get things I want.”), and Fun‐Seeking (e.g., “I am always willing to try something new if I think it will be fun.”). Individual differences in these scales map to clinical outcomes in the realm of anxiety and impulsivity, respectively. Items within these scales have high internal reliability (all α ≥ 0.66). The BIS/BAS also demonstrates strong convergent and divergent validity with other measures of motivation, emotion processing, extraversion/introversion, and personality (see Table 2 in Carver and White 1994). In the current study, BIS/BAS Scale outcomes were used to explore individual differences in our sample as they relate to performance on the BELT.

### 2.3 Procedures

All procedures were approved by the Institutional Review Boards (IRBs) at Assumption University and Merrimack College. Undergraduate research participants were recruited through SONA. All participants completed a prescreening survey to determine eligibility (described above).

Research assistants first collected written informed consent. Participants were reminded that they could ask questions or withdraw from the study at any time. After consent was obtained, participants completed the BIS/BAS Scale on an iPad (untimed). Participants then completed the BELT. The BELT took approximately 20 minutes to complete. After completion of the BELT, participants were dismissed from the study protocol.

### 2.4 Data Analysis

Wilcoxon signed‐rank tests were used to test for gender differences in total adjusted pumps and total number of explosions across the entire BELT task to determine whether gender needed to be included as a covariate in the planned statistical models. Pearson correlations were computed to explore (i) the association between early exploratory behavior, as measured by the total adjusted pumps during the first task block (i.e., first ⅓ of trials), and total points accumulated across the task and (ii) relations between total adjusted pumps across the entire task, as a measure of successful risk‐taking, and scales from the BIS/BAS.

Generalized linear models (GLM, lme4 package in R) were used to evaluate relations between (i) the number of explosions across task blocks (block 1: first ⅓ of trials, block 2: middle ⅓ of trials, and block 3: last ⅓ of trials), (ii) the number of adjusted pumps by balloon type (variable, stable long, stable short) and task block, (iii) the performance (i.e., total pumps) on the next balloon by balloon type and outcome on the previous balloon (i.e., explosion or reward collected), and (iv) the difference between the number of adjusted pumps and the optimal number of pumps by balloon type and task block. Balloon type and task block were entered in the GLM as fixed effects and subject was entered as a random effect. Models for “number of balloon pumps” and “number of explosions” as the outcome variable were modeled using the glmer function and family = poisson. Effect sizes for significant effects were reported as the rate ratio with 95% confidence interval (CI). The significance level was set at *p* = 0.05 for all analyses. The Holm–Bonferroni method was applied to adjust for multiple comparisons in the correlational analyses.

## 3 Results

There were no significant differences by gender for either total adjusted pumps (*W* = 257.5, *p* = 0.68) or total explosions (*W* = 268, *p* = 0.83) across the task; therefore, gender was not included as a covariate in any of the subsequent analyses.

### 3.1 Risk‐Taking Supports Implicit Learning

**FIGURE 2:**
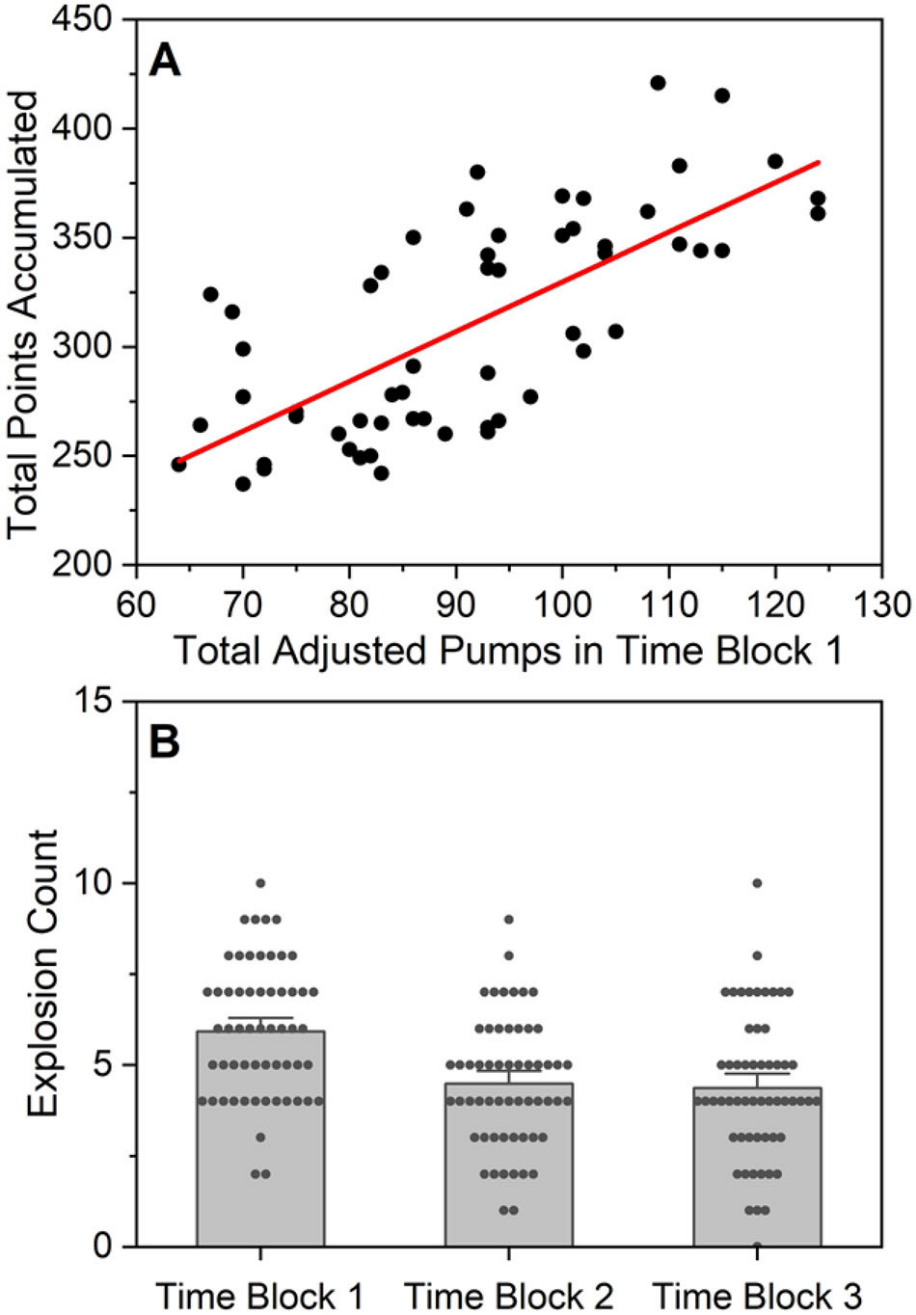
(A) Positive association between total adjusted pumps in Block 1 (the first ⅓ of the trials) and the total points accumulated across the task. (B) The number of explosions decreased across the task. Individual points represent the average number of explosions for each participant within each block. Bar height represents the overall mean across participants for each Balloon Type x Block. Error bars represent standard errors.

There was a significant positive relationship between the total adjusted pumps during block 1 (first ⅓ of trials) and the total points accumulated across the task (*r* = 0.71, *p* < 0.001; Figure 2A). Furthermore, in the model evaluating the number of explosions by task block (block 1: first ⅓ of trials, block 2: middle ⅓ of trials, and block 3: last ⅓ of trials), there was a significant main effect of block (𝜒^2^ = 10.10, *p* = 0.006) such that the number of explosions was significantly lower during both block 2 (rate ratio = 0.85, 95% CI = 0.72‐1.00, *p* = 0.05) and block 3 (rate ratio = 0.77, 95% CI = 0.66‐0.91, *p* = 0.002) compared to block 1 (Figure 2B); there was no difference in the number of explosions between block 2 and block 3 (rate ratio = 0.9, 95% CI *=* 0.76‐1.08). Together, these results indicate that exploratory risk‐taking behavior early in the task supports performance later in the task.

**FIGURE 3:**
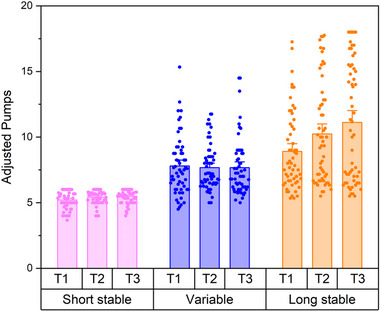
Adjusted pumps for Balloon Types (short stable, variable, or long stable) by Task Block (T1 = first ⅓ of trials, T2 = second ⅓ of trials, T3 = third ⅓ of trials). Individual points represent the average for each participant within each Balloon Type x Task Block. Bar height represents the overall mean across participants for each Balloon Type x Task Block. Error bars represent standard errors.

In the model evaluating changes in adjusted pumps by balloon type (variable, stable long, stable short) and task block, there was a significant main effect of balloon type on the adjusted pumps per balloon (𝜒^2^ = 159.02, *p* < 0.001) and a significant interaction between balloon type and block (𝜒^2^ = 34.06, *p* < 0.001) but no significant main effect of block (𝜒^2^ = 1.08, *p* = 0.58). Compared to balloons with stable, short explosion rates, participants made significantly more pumps on balloons with variable explosion rates (rate ratio = 1.48, 95% CI = 1.35‐1.61, *p* < 0.001) and stable, long explosion rates (rate ratio = 1.68, 95% CI *=* 1.55‐1.83, *p* < 0.001; Figure 3). Based on post‐hoc comparisons, the significant interaction effect was due to an increase in the number of pumps on balloons with long, stable explosion rates over blocks of the task (block 1 to block 2: rate ratio = 1.11, 95% CI = 1.00‐1.24, *p* = 0.05; block 1 to block 3: rate ratio = 1.18, 95% CI = 1.05‐1.31, *p* = 0.002).

**FIGURE 4:**
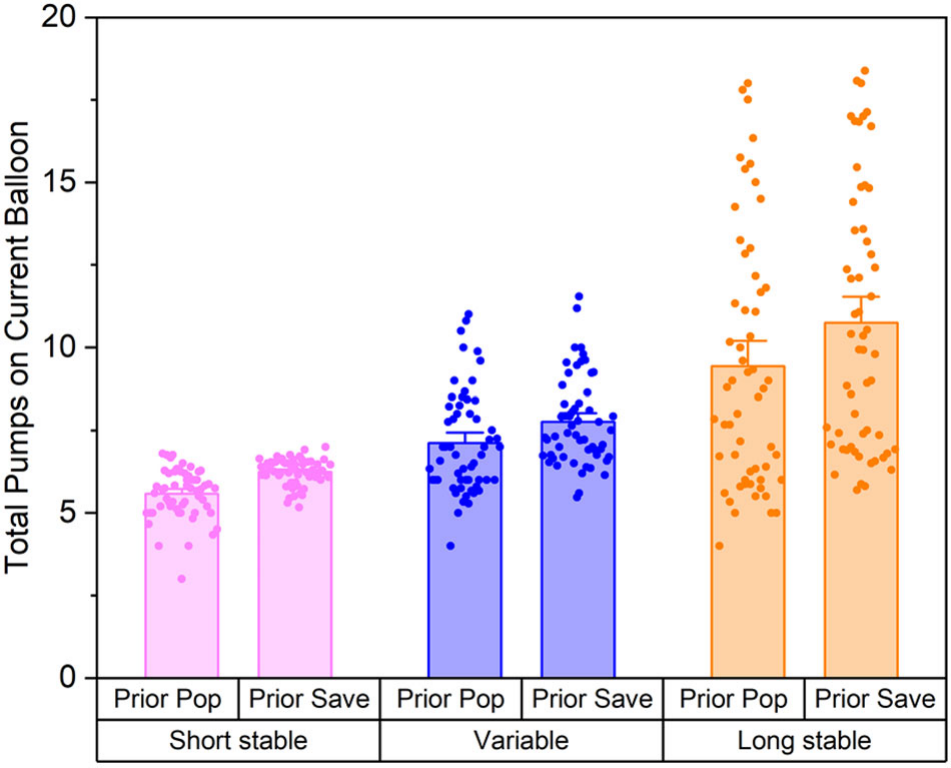
Sensitivity to negative feedback: outcome on previous balloon and performance on the subsequent balloon. Individual points represent the average total pumps for each participant within each balloon type following each outcome (pop or save). Bar height represents the overall mean across participants for each Balloon Type x Outcome. Error bars represent standard errors.

To evaluate sensitivity to negative feedback, another model was generated to evaluate the relation between outcome on the previous balloon (explosion or saved points) and performance (total pumps) on the next balloon by balloon type. There was a significant main effect of the previous outcome (𝜒^2^ = 20.29, *p* < 0.001) and the current balloon type (𝜒^2^ = 310.01, *p* < 0.001) but no significant interaction effect (𝜒^2^ = 0.88, *p* = 0.64) on the number of pumps on the current balloon (Figure 4). There was a significant increase in the number of pumps following an outcome of saved points compared to an explosion (rate ratio = 1.14, 95% CI = 1.08‐1.21, *p* < 0.001) and a significant increase in pumps if the current balloon type was variable (rate ratio = 1.28, 95% CI = 1.20‐1.37, *p* < 0.001) or stable, long (rate ratio = 1.72, 95% CI = 1.62‐1.83, *p* < 0.001) compared to stable, short.

**FIGURE 5:**
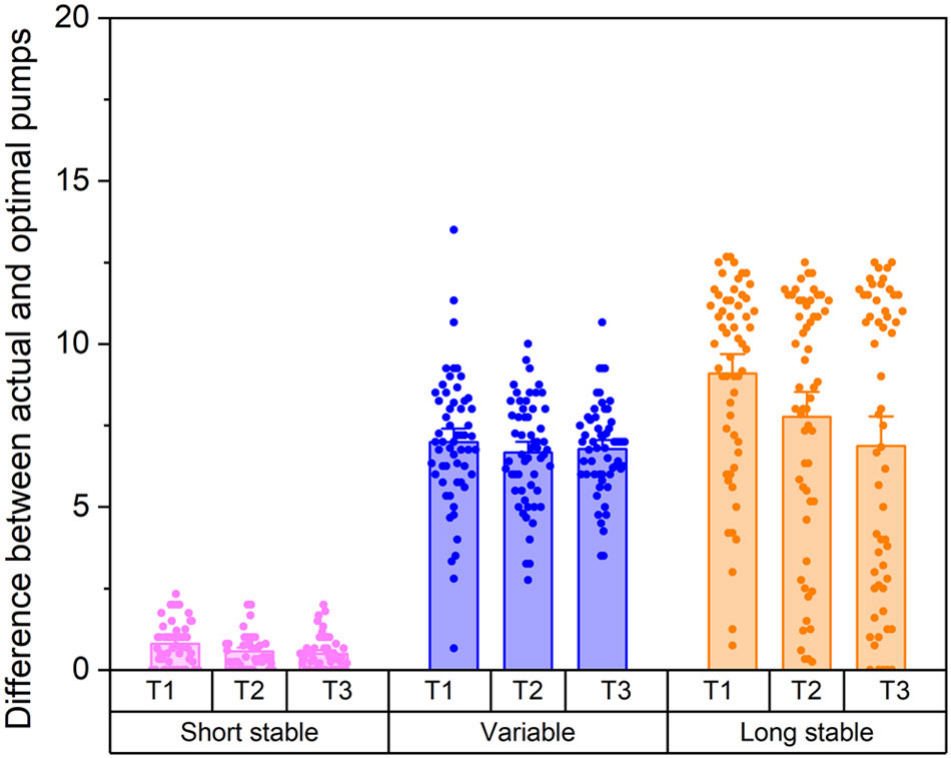
Difference between actual and optimal pumps across task blocks. Individual points represent the average difference for each participant within each Balloon Type x Block. Bar height represents the overall mean across participants for each Balloon Type x Block. Error bars represent standard errors.

A final model was generated to evaluate the difference between the number of adjusted pumps and the optimal number of pumps (i.e., balloon durability) by balloon type across task block. There was a significant main effect of block (𝜒^2^ = 19.47, *p* < 0.001) and balloon type (𝜒^2^ = 726.71, *p* < 0.001), and a significant interaction effect between block and balloon type (𝜒^2^ = 34.46, *p* < 0.001; Figure 5). The difference between the number of adjusted pumps and the optimal number of pumps decreased significantly from block 1 to block 2 (rate ratio = 0.65, 95% CI = 0.50‐0.84, *p* < 0.001) and from block 1 to block 3 (rate ratio = 0.59, 95% CI = 0.46‐0.76, *p* < 0.001), providing evidence that participants learned the optimal number of pumps across the task. The difference between the number of adjusted pumps and the optimal number of pumps was significantly higher on balloons with variable explosion rates (rate ratio = 7.87, *95 % CI* = 6.58‐9.42, *p* < 0.001) and on balloons with stable, long explosion rates (rate ratio = 10.56, 95% CI = 8.85‐12.59, *p* < 0.001) compared to balloons with stable, short explosion rates, reflecting the increased number of pumps possible on these balloon types.

### 3.2 Risk‐Taking Positively Relates to Persistent Pursuit of Goals

**FIGURE 6:**
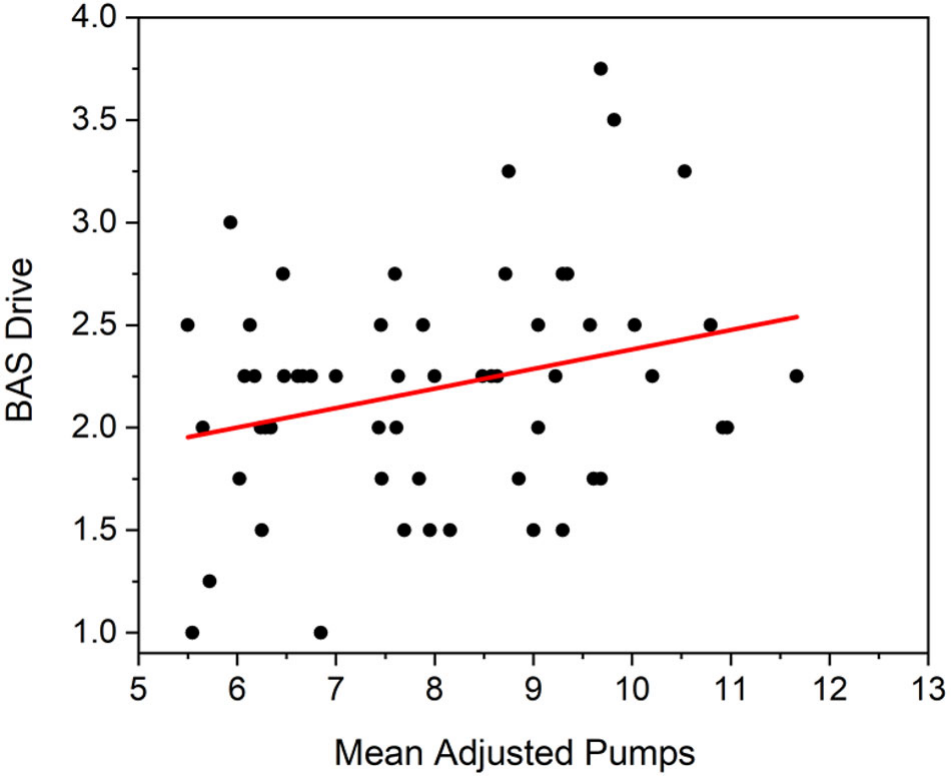
Correlations between risk‐taking outcomes and persistent pursuit of goals as measured by BAS Drive. BAS = Behavioral Activation System.

To evaluate relations between risk‐taking and individual differences in motivation and emotional processing, bivariate correlations were computed between the total adjusted pumps on the BELT and the BIS/BAS Scales. The total number of adjusted pumps on the BELT was significantly, positively correlated with the BAS Drive Scale (*n* = 57, *r* = 0.277, *p* = 0.037; Figure 6) but was not correlated with the BAS Fun Seeking Scale (*r* = 0.098, *p* = 0.470), BAS Reward Response Scale (*r* = ‐0.053, p = 0.70), or BIS Scale (*r* = 0.135, *p* = 0.316). Notably, however, when *p* values were adjusted for multiple comparisons, the correlation between total number of adjusted pumps and the BAS Drive Scale was no longer significant (*p* = 0.149, Holm–Bonferroni method).

## 4 Discussion

The primary aim of this study was to evaluate risk‐related learning in neurotypical young adults. Consistent with our hypothesis and the work of others (Humphreys et al. 2013), our results indicate that early, exploratory risk‐taking supported learning in our sample. Early exploration, measured by the total adjusted number of pumps in the first third of the task, was associated with accumulating more total points across the task (Figure 2A). Moreover, the number of explosions decreased across the task (Figure 2B) and participants increased the number of times they pumped the safer balloons with stable, long explosion rates over the course of the task (Figure 3). Given that there was no explicit feedback on task performance (other than points earned and balloon explosions), this suggests that risk‐taking supported implicit learning in our sample as participants learned which balloons were safer and, consequently, more advantageous for task success. Relatedly, the difference between the number of pumps and the optimal number of pumps (i.e., balloon durability) decreased across the task, suggesting that participants not only learned which balloons were safer but started to learn how many pumps were possible before the balloon would explode (Figure 5).

Humphreys et al. (2013)’s sample demonstrated a consistent number of pumps across blocks for both stable conditions (short and long) and a decrease in the pumps for the variable condition. In contrast, our young adult sample significantly increased the number of pumps on long, stable balloons across blocks. These differences may be due to the number of trials administered in each study, as we doubled the number of trials (54 trials) that Humphreys’ study used (27 trials). A larger number of trials may have provided a greater opportunity for learning. Nonetheless, both instances provide evidence of learning, as these changes in behavior ultimately improved overall task performance.

Our data also indicate that participants utilized feedback from previous trials to improve future task performance. This is evidenced by the increased number of pumps observed following trials when a balloon did not explode (e.g., points were saved, indicating successful risk‐taking) versus trials when a balloon exploded (e.g., balloon was overinflated, unsuccessful risk‐taking; Figure 4). Reduced pumps after explosions represent sensitivity to negative feedback. Consistent with Humphreys et al. (2013, 2011), this finding suggests that the knowledge of outcomes from earlier risks shaped future behaviors which, ultimately, improved task outcomes.

Together, these results suggest that context‐dependent risk‐taking supports learning—an adaptive outcome—in young adults. This work extends a growing body of literature outlining negative implications for risk‐taking (Boyer 2006; Gong et al. 2022; Mishra et al. 2010; Petry 2001; Secades‐Villa et al. 2016) and supports theoretical perspectives of adaptive risk‐taking in adolescence (Duell and Steinberg 2019; Ellis et al. 2012). Ellis et al. 2012 argue that risk‐taking behavior may serve evolutionary advantages during critical developmental transitions such as adolescence. For example, aggressive behaviors may signal adaptive traits such as bravery or social status. Following this framework, Ellis et al. (2012) propose that interventions should promote social pathways by cultivating environmental conditions that reduce stress, increase predictability, and remove harsh dynamics (e.g., “zero tolerance” policies) to match adolescents’ goals and motivations rather than working against their instincts. Whether or not such suggestions improve risk‐related outcomes in adulthood—a critical developmental transition when additional social challenges arise—warrants additional research.

The second aim of our study was to determine how risk‐taking outcomes would correlate to individual differences in motivation and emotional processing (as measured by BIS/BAS Scales). Our hypothesis was supported, and was consistent with prior research (Cooper et al. 2000; Leikas et al. 2009; Li et al. 2019), as risk‐taking (BELT total adjusted pumps) was positively correlated to persistent pursuit of goals (as measured by the BAS Drive Scale; Figure 6). The BAS Drive Scale is based on participant self‐report on 4‐items that measure agreement with statements related to motivation for goal‐directed behavior including *“I go out of my way to get things I want,” “When I want something I usually go all‐out to get it,” “If I see a chance to get something I want I move on it right away,”* and *“When I go after something I use a ‘no holds barred’ approach.”*

This finding has compelling implications as goal‐directed behaviors strongly predict academic success (Eppler and Harju 1997; Steinmayr et al. 2011), particularly in non‐traditional students who take time off during undergraduate studies (Eppler and Harju 1997). Importantly, however, these results should be interpreted with caution, as the effect was no longer significant after controlling for multiple comparisons. A post hoc power analysis (G*Power 3.1.9.7) indicated that we only had 74% and 73% power to detect a significant correlation at an alpha level of 0.05 between for BELT total pumps and total adjusted pumps and the BAS Drive Scale. As such, research with a larger, more diverse sample is needed to further explore relations between risk‐taking and motivation and emotional processing in adults. Additionally, future research is needed to determine how risk‐related learning may support academic outcomes in diverse bodies of college students, as academic outcomes were not evaluated in the current study. Moreover, neuroimaging work is needed to understand how the development and activity of neural systems—such as those posed in Ernst's Triadic Model (Ernst et al. 2006; Ernst 2014)—influences risk‐taking behaviors and relations with motivation and emotional processes in adults.

Although underpowered, our correlational results may be of interest to individuals in educational or professional settings where risk‐related operations affect performance, physical safety (e.g., military, healthcare settings), financial gain or stability (e.g., investment banking, entrepreneurship), and creativity/innovation (e.g., artists, writers, filmmakers, technology, research and design) (Bechara et al. 1997; Breivik et al. 2019; Giaccone and Magnusson 2022; Macko and Tyszka 2009; McGowan 2007). In these careers and others, successful assessment and management of risk relates to improved outcomes.

## 5 Conclusions

The current study was innovative as we used an objective measure of risk‐taking behavior to demonstrate risk‐related learning in adults. These findings corroborate prior research which indicates that risk‐taking supports learning (Humphreys et al. 2013). Additionally, risk‐taking tendencies were positively related to goal‐driven behavior in our sample. Together, these findings suggest that context‐dependent risk‐taking is adaptive and may improve learning‐based educational and professional outcomes. Results obtained from this study are expected to inform interventions that shape adaptive outcomes during this critical developmental period.

## Author Contributions

**Amanda Cremone‐Caira**: conceptualization; writing–original draft, methodology; writing–review and editing; project administration; investigation. **Melissa St. Hilaire**: writing–review and editing; formal analysis; visualization.

## Conflicts of Interest

The authors declare no conflicts of interest.

## Ethics Approval

All procedures were approved by the Institutional Review Boards (IRBs) at Assumption University and Merrimack College.

## Consent

Written, informed consent was obtained from all participants prior to data collection.

### Peer Review

The peer review history for this article is available at https://publons.com/publon/10.1002/brb3.70409.

## Data Availability

The de‐identified data that support the findings of this study are openly available on the Open Science Framework (OSF) at https://osf.io/4ugc9/files/osfstorage/67bddf4f8304ea08a39fb064.

## Endnotes

1 The research study design and analyses described were not preregistered in an independent, institutional registry.

2 Information pertaining to age and gender was missing from one participant and race was missing from three participants.
